# Synthesis and Characterization ofTriphenylphosphine Adducts of Ferrocene-Based Palladacycles and Their Performance in the Suzuki and Sonogashira Reactions with Bromo- and Chloroarenes

**DOI:** 10.3390/molecules17055532

**Published:** 2012-05-09

**Authors:** Su-Zhen Bai, Chen Xu, Hong-Mei Li, Zhi-Qiang Wang, Wei-Jun Fu

**Affiliations:** 1 College of Chemistry and Chemical Engineering, Pingdingshan University, Pingdingshan, Henan 467002, China; 2 College of Chemistry and Chemical Engineering, Luoyang Normal University, Luoyang, Henan 471022, China

**Keywords:** adduct, palladacycle, crystal structure, Suzuki reaction, Sonogashira reaction

## Abstract

A new triphenylphosphine adduct of cyclopalladated ferrocenylpyridazine containing a chloride anion, **2a**, has been synthesized from the reaction of the chloride-bridged palladacyclic dimer **1a** with triphenylphosphine. The corresponding adducts **3a**,**b** containing iodide anion have been readily prepared through anion exchange reactions of **2a**,**b** with NaI in acetone. These complexes were characterized by elemental analysis, IR and ^1^H-NMR. Additionally, their crystal structures have been determined by X-ray diffraction and intermolecular C–H···X (Cl, Br, I) bonds were found in the crystals. The use of these palladacycles as catalysts for the Suzuki and Sonogashira reactions was examined. The complexes **2a**,**b** exhibited higher catalytic activity than the corresponding **3a**,**b** in the Suzuki reaction. However, the order of activity of adducts with varying halogen anions is **3a**~**3b** > **2a**~**2b** in the Sonogashira reaction.

## 1. Introduction

Palladium-catalyzed coupling reactions such as the Suzuki and Sonogashira reactions have become an extremely powerful method for the formation of carbon-carbon bonds in organic synthesis [1,2,3]. A number of Pd catalyst precursors, usually simple palladium salts or complexes associated with appropriate ligands, can catalyse these reactions under various reaction conditions. Among them, palladacycles have been extensively applied in coupling reactions as effective catalyst precursors, due to their ready preparation and modification, high activity and relative stability [4,5,6]. In the cyclopalladation reactions, owing to the poor solubility of the dimers, they are usually subjected to a bridge-splitting reaction with monophosphines to produce the corresponding adducts. 

**Scheme 1:**
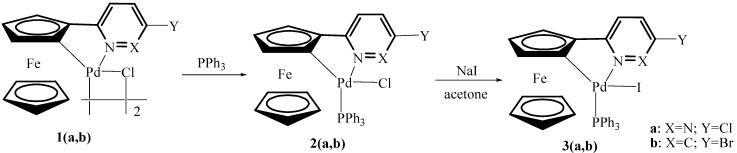
Synthesis of triphenylphosphine adducts of palladacycles **2**–**3**.

In recent years, we have reported a variety of cyclometalated complexes containing N-donor ferrocenyl ligands and found that palladacycle adducts were far more active than the corresponding dimers in coupling reactions [7,8,9]. In view of these findings and our continuing interest in the synthesis of palladacycles and the applications of these systems, we have prepared a new chloride anion-containing palladacycle adduct **2a** and two corresponding adducts **3a**,**b** containing iodide anion(Scheme 1). Their application to the Suzuki and Sonogashira reactions were investigated since we were interested to see the effect of halogen anions on the catalytic activity. The results are described in this paper.

## 2. Results and Discussion

### 2.1. Synthesis and Characterization of Complexes ***2–3***

The chloride-bridged palladacyclic dimers **1a**,**b** were prepared according to the published procedures [10,11]. Then each of them was treated with 1.1 equivalent of triphenylphosphine per palladium in CH_2_Cl_2_ at room temperature for 30 min to afford the corresponding adducts **2a**,**b** as red solids. The iodide anion-containing palladacycle adducts **3a**,**b** have been readily prepared through anion exchange reactions of **2a**,**b** with NaI in acetone (Scheme 1). These complexes are air- and moisture-stable, both in the solid state and in solution. They are very soluble in chloroform, dichloromethane and acetone, but insoluble in petroleum ether and *n*-hexane. These complexes were characterized by elemental analysis, IR and ^1^H-NMR. They exhibit similar peaks for the Cp ring, with a proton ratio of 1:1:1:5, clearly showing that they are both *ortho*-cyclopalladated complexes. In general terms, the spectra of **2a**,**b** were similar to those of the corresponding **3a**,**b**. In order to further investigate the structures of these complexes and the effect of halogen anions on the structure, their crystal structures have been determined by X-ray diffraction.

### 2.2. Crystal Structures of the Complexes ***2–3***

**Figure 1:**
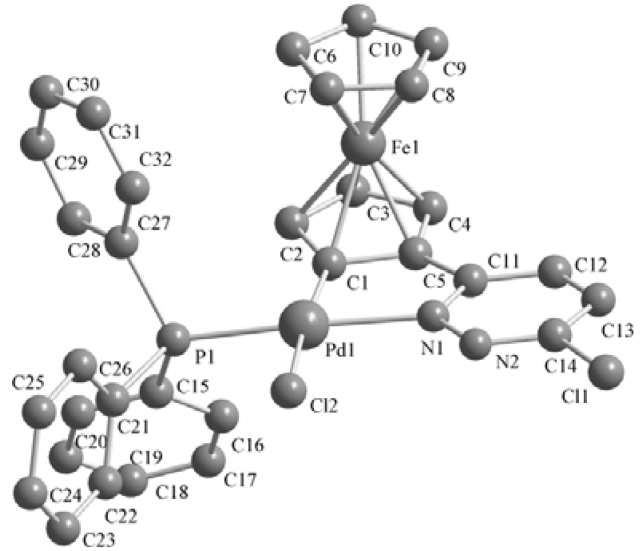
Molecular structure of complex **2a**. H atoms are omitted for clarity.

**Figure 2:**
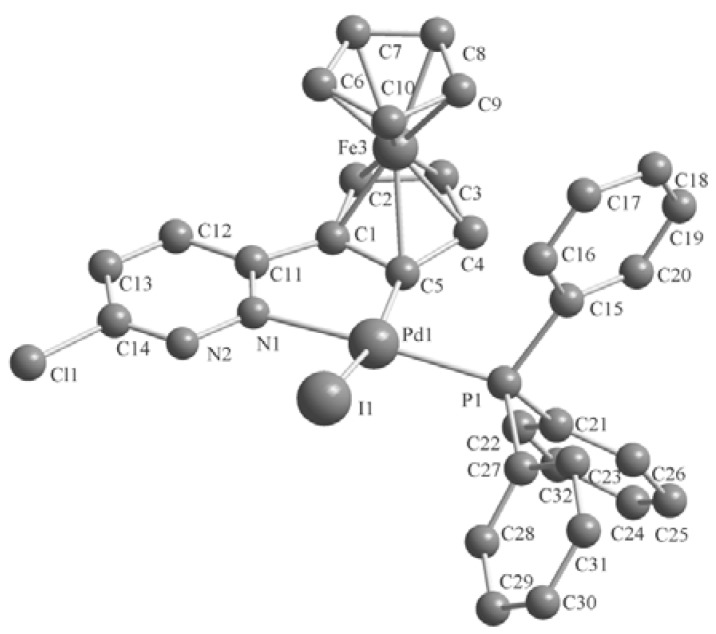
Molecular structure of complex **3a**. H atoms are omitted for clarity.

**Figure 3:**
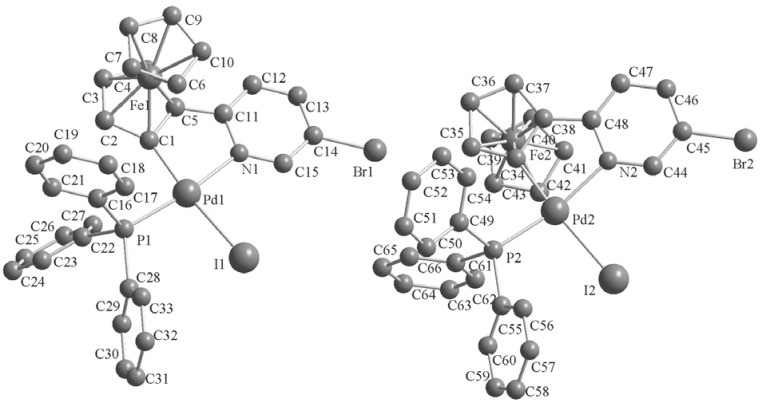
Molecular structure of complex **3b**. H atoms are omitted for clarity.

Single crystals of these complexes were obtained by crystallization from CH_2_Cl_2_-petroleum ether at room temperature. The molecular structures are shown in Figure 1, Figure 2, Figure 3. The crystal structure of **3b** reveals two molecules in the asymmetric unit with different geometry (corresponding values for the second structure are given in brackets). Selected bond lengths and angles are listed in Table 1. The single-crystal X-ray analysis reveal that all complexes display *trans* configurations in the solid state. The Pd atom in each cyclopalladated unit is in a slightly distorted square-planar environment, being coordinated to phosphorus, chlorine (iodine), nitrogen and the carbon atom of the ferrocenyl moiety. The Pd–N [2.144(7)–2.160(6) Å] and Pd–P [2.236(2)–2.246(2) Å] bond lengths of **3a**,**b** are similar to those of the corresponding **2a**,**b** [2.140(3)–2.1418(19) and 2.2322(11)–2.2369(7) Å], while Pd–I [2.6491(10)–2.6752(12) Å] bond lengths of **3a**,**b** are obviously longer than the Pd–Cl [2.3393(8)–2.3642(2) Å] bond lengths in the corresponding adducts **2a**,**b** [11].

**Table 1:** Selected bond lengths (Å) and angles (°) for complexes **2a***–***3b**.

Compound	2a	3a	3b
Pd(1)-C[(1) or (5)]	1.995(2)	2.003(9)	1.997(7)[2.011(8)]
Pd(1)-N(1)	2.1418(19)	2.144(7)	2.160(6)[2.160(6)]
Pd(1)-P(1)	2.2369(7)	2.246(2)	2.238(2)[2.236(2)]
Pd(1)-[Cl(2) or I(1)]	2.3393(8)	2.6752(12)	2.6491(10)[2.6574(10)]
C[(1) or (5)]-Pd(1)-N(1)	80.87(9)	80.3(3)	81.0(3)[81.1(3)]
C[(1) or (5)]-Pd(1)-P(1)	92.30(8)	91.8(3)	90.2(2)[91.0(2)]
N(1)-Pd(1)-[Cl(2) or I(1)]	92.94(6)	94.79(19)	93.46(17)[93.23(17)]
P(1)-Pd(1)-[Cl(2) or I(1)]	94.17(3)	95.20(6)	95.52(6)[96.40(6)]

**Figure 4:**
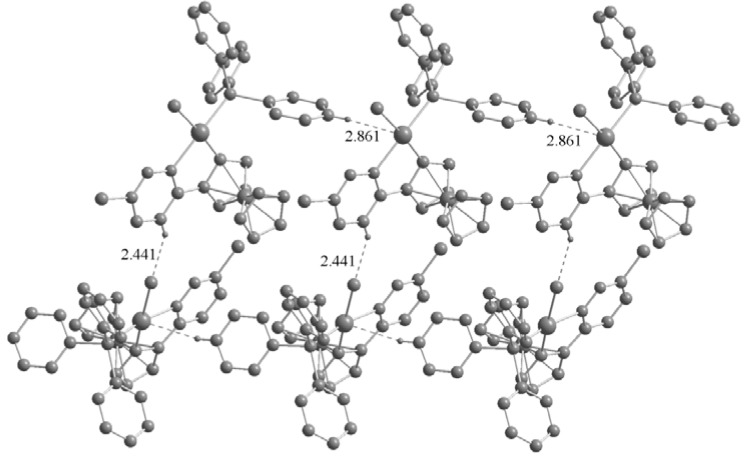
Two-dimensional network structure of complex **2a** formed by C–H···Cl and C–H···Pd hydrogen bonds. Non-hydrogen bonding H atoms are omitted for clarity.

**Figure 5:**
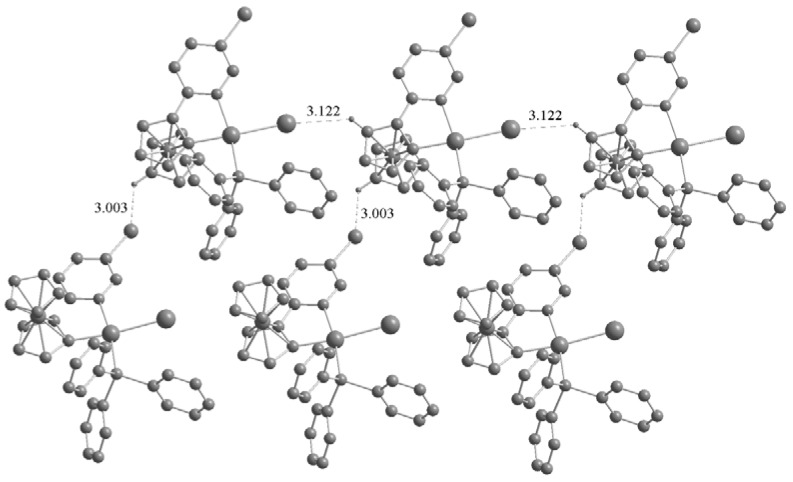
One-dimensional chain structure of complex **3b** formed by C–H···I(Br) hydrogen bonds. Non-hydrogen bonding H atoms are omitted for clarity.

The most striking common feature of the structures of the three palladacycles is the intermolecular C–H···X (Cl, Br, I) hydrogen bonds (Figure 4 and Figure 5). The C–H···X–M hydrogen bonding can be used to assemble transition metal based building blocks into supramolecular structures since the H-bonding acceptor capability of terminal metal-bound chlorine (M–X) is stronger than their C–X analogues in organometallic compounds [12,13]. Basically, the crystal structures of the two complexes **2a** and **3a** are quite similar, with chlorine (iodine) anion forming C–H···X(Cl, I) hydrogen bonds (Cl···H = 2.441 and I···H = 3.061 Å), however, the chlorine atom in the pyridazine ring does not participate in hydrogen bonding. It is noteworthy that intermolecular C–H···Pd hydrogen bonds are present in the crystals of **2a** (Pd···H = 2.861 Å) and **3a** (Pd···H = 2.736 Å). Although these interactions have been extensively studied [14], there are a few reports concerning palladacycles [15,16,17]. Owing to the C–H···X(Cl, I) and C–H···Pd hydrogen bonds, the crystal structures of the **2a** and **3a** are extended to a 2D network architecture (Figure 4). Unlike **2a** and **3a**, the complex **3b** has a one-dimensional chain structure formed by C–H···I hydrogen bonds between an iodine atom and the adjacent C–H group of the Cp ring. This is different from that of **2b** which presents C–H···Cl hydrogen bonds between a chlorine atom and the adjacent C–H group of the pyridine ring [11]. In addition, C–H···Br hydrogen bonds between bromine atom and the adjacent C–H group of Cp ring are also present in the crystal of **3b** (Figure 5). 

### 2.3. Application in Suzuki Reaction

Phosphine adducts of palladacycles have been successfully used as precatalysts in Suzuki coupling reactions [4,5]. The active species was proposed to be a Pd(0) “Pd-PR_3_” species and it was shown that the cyclopalladated ligand was released from the metal center during activation of the Pd(II) precatalyst [18,19]. Usually, cyclopalladated ligands have little effect on the catalytic activity, however, variation of the anionic ligand X(Cl, Br, I, OAc, *etc*.) is found to have some effect on the catalytic activity [20,21]. In order to further investigate the effect of halogen anions on the catalytic activity, we studied the Suzuki reactions catalyzed by the palladacycle adducts **2**–**3** containing chloride/iodide anions. 

In the present study, our initial exploration of the reaction focused on the coupling of 4-bromotoluene with phenylboronic acid. Based on our previous experiments with palladacylic precatalysts for Suzuki coupling reactions [11,17,22], the reaction was performed under nitrogen atmosphere in dioxane in the presence of Cs_2_CO_3_ as base at 100 °C for 12 h, affording the coupling product in good yields (Table 2, entries 1–4). For an *ortho*-substituent such as 2-bromotoluene, the yields decreased slightly (entries 5–8). The complexes **2a**,**b** exhibited higher catalytic activity than those of the corresponding **3a**,**b** under the same reaction conditions. Even *o*-bromoanisole provide good yields by using 0.5 mol% of **2a**,**b** (entries 9–10). For electron-deficient aryl bromides such as 4-bromonitrobenzene, they could be coupled very efficiently with a catalytic loading as low as 0.1 mol% (entries 11–14). We next investigated Suzuki coupling of 4-chlorotoluene under the same reaction conditions. In this case, however, the complexes **2a**,**b** were almost inactive (entries 15–16). It can be seen from the above results that the activity of adducts in the Suzuki reaction falls in the order **2a**~**2b** > **3a**~**3b**, and the halogen anions have some effect on the catalytic activity, which is consistent with the literature [18,21].

**Table 2:** Suzuki coupling of aryl halides with phenylboronic acid catalysed by complexes **2** and **3** ^a^. 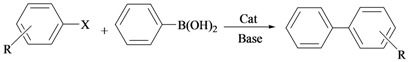

Entry	X	R	Catalyst (mol% Pd)	Yield ^b^ (%)
1	Br	*p*-CH_3_	**2a** (0.5)	93
2	Br	*p*-CH_3_	**2b** (0.5)	95
3	Br	*p*-CH_3_	**3a** (0.5)	84
4	Br	*p*-CH_3_	**3b** (0.5)	82
5	Br	*o*-CH_3_	**2a** (0.5)	91
6	Br	*o*-CH_3_	**2b** (0.5)	92
7	Br	*o*-CH_3_	**3a** (0.5)	73
8	Br	*o*-CH_3_	**3b** (0.5)	75
9	Br	*o*-OCH_3_	**2a** (0.5)	88
10	Br	*o*-OCH_3_	**2b** (0.5)	86
11	Br	*p*-NO_2_	**2a** (0.1)	98
12	Br	*p*-NO_2_	**2b** (0.1)	97
13	Br	*p*-NO_2_	**3a** (0.1)	92
14	Br	*p*-NO_2_	**3b** (0.1)	90
15	Cl	*p*-CH_3_	**2a** (1)	trace
16	Cl	*p*-CH_3_	**2b** (1)	trace

^a^ Reaction conditions: aryl halides (0.5 mmol), PhB(OH)_2_ (0.75 mmol), Cs_2_CO_3_ (0.75 mmol), dioxane (3 mL), 100 °C, 12 h; ^b^ Isolated yields (average of two experiments).

### 2.4. Application in Sonogashira Reaction

The high activity of triphenylphosphine adducts of palladacycles **2**–**3** in the Suzuki coupling of aryl bromides encouraged us to examine their activity and the effect of halogen anions in the Sonogashira reaction. Usually, the Sonogashira reaction is mediated by a palladium catalyst with copper cocatalyst and amine base. However, many phosphine-palladium complexes are sensitive to both air and moisture, and are both costly and toxic. Moreover, the use of a copper reagent results in contamination of the coupling products with metal residues. Thus a lot of efforts are underway toward developing new palladium catalysts and copper- and amine-free conditions for the Sonogashira reaction [23,24,25,26]. Among them, a wide variety of palladacycles have been reported and successfully used in the Sonogashira reaction [5,27,28,29,30]. To the best of our knowledge, there was no report concerning the effect of halogen anions on the catalytic activity of palladacycle for the Sonogashira reaction.

The adducts of palladacycles **2**–**3** were evaluated in arylation reaction of phenylacetylene with aryl halides under copper- and amine-free conditions (Table 3). The model reaction of bromobenzene with phenylacetylene produced maximum yields when CsOAc was used as a base, dimethylacetamide (DMA) as a solvent, and the reaction occurred at 120 °C for 24 h. 

**Table 3:** Sonogashira reaction of aryl halides with phenylacetylene catalysed by complexes **2** and **3** ^a^. 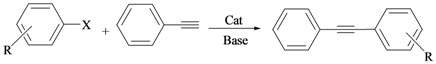

Entry	X	R	Catalyst (mol% Pd)	Yield ^b^ (%)
1	Br	H	**2a** (1)	88
2	Br	H	**2b** (1)	90
3	Br	H	**3a** (1)	96
4	Br	H	**3b** (1)	97
5	Br	*o*-CH_3_	**2a** (1)	81
6	Br	*o*-CH_3_	**2b** (1)	80
7	Br	*o*-CH_3_	**3a** (1)	92
8	Br	*o*-CH_3_	**3b** (1)	94
9	Br	*o*-CH_3_	**3a** (1)	89
10	Br	*o*-CH_3_	**3a** (1)	87
11	Br	*p*-COCH_3_	**2a** (0.1)	91
12	Br	*p*-COCH_3_	**2b** (0.1)	92
13	Br	*p*-COCH_3_	**3a** (0.1)	96
14	Br	*p*-COCH_3_	**3b** (0.1)	98
15	Cl	*p*-COCH_3_	**3a** (2)	35
16	Cl	*p*-COCH_3_	**3b** (2)	37
17	Cl	*p*-NO_2_	**3a** (2)	46
18	Cl	*p*-NO_2_	**3b** (2)	49

^a^ Reaction conditions: aryl halides (0.5 mmol), arylacetylene (0.6 mmol), CsOAc (0.75 mmol), DMA (3 mL), 120 °C, 24 h; ^b^ Isolated yields (average of two experiments).

All the tested palladacycle precatalysts **2**–**3** showed good activity for the above reaction, giving the coupled product in 88–97% yields (entries 1–4). It was interesting that the complexes **3a**,**b** exhibited higher catalytic activity than those of the complexes **2a**,**b** in the Sonogashira reaction. This is different from Suzuki reaction. Then, we investigated the Sonogashira coupling of 2-bromotoluene under the same conditions to further compare the effect of halogen anions on the catalytic activity (entries 5–8). Similar to the results of bromobenzene, the activity of adducts falls in the order **3a**~**3b** > **2a**~**2b**. For electron-rich *o*-bromoanisole, the yields decreased slightly, but still were very high (89% and 87%, entries 9–10). In the case of activated 4-bromoacetophenone, it was not surprising that good yields were obtained with a catalytic loading as low as 0.1 mol% (entries 11–14). Furthermore, Sonogashira reaction of phenylacetylene with aryl chlorides were also studied in this system. For activated chlorides such as 4-chloroacetophenone and 4-chloronitrobenzene, the coupled products could be only produced in 35–49% yields by using 2 mol% of **3a**,**b** (entries 15–18). 

## 3. Experimental

### 3.1. General Procedures

Solvents were dried and freshly distilled prior to use. All other chemicals were commercially available expect for the chloride-bridged palladacyclic dimers **1a**,**b** and **2b** were prepared according to published procedures [10,11]. All other chemicals were used as purchased. Elemental analyses were determined with a Thermo Flash EA 1112 elemental analyzer. IR spectra were collected on a Bruker VECTOR22 spectrophotometer using KBr pellets. ^1^H-NMR spectra were recorded on a Bruker DPX-400 spectrometer in CDCl_3_ with TMS as an internal standard. 

### 3.2. The Synthesis of Complex ***2a***

A solution of chloride-bridged palladacyclic dimer **1a** (0.1 mmol) and triphenylphosphine (0.22 mmol) in CH_2_Cl_2_ (10 mL) was stirred at room temperature for 30 min. The product was separated by passing through a silica gel column with dichloromethane as eluent. The first band was collected to afford complex **2a** after the evaporation of the solvent. [PdCl{[(η^5^-C_5_H_5_)]Fe[(η^5^-C_5_H_3_)-N_2_C_4_H_2_-Cl]}PPh_3_] (**2a**): Yield 90%. Found (%): C, 55.1; H, 3.4; N, 4.2. Calc. (%) for C_32_H_25_Cl_2_FeN_2_PPd: C, 54.8; H, 3.6; N, 4.0. IR (KBr, cm^-1^): 3045, 1584, 1533, 1472, 1435, 1392, 1376, 1273, 1161, 1097, 999, 855, 815, 743, 689. ^1^H-NMR (CDCl_3_): δ 7.84–7.80 (m, 6H, ArH), 7.40–7.33 (m, 11H, ArH), 4.65 (s, 1H, C_5_H_3_), 4.22 (s, 1H, C_5_H_3_), 3.75 (s, 5H, C_5_H_5_), 3.43 (s, 1H, C_5_H_3_). 

### 3.3. General Method for the Synthesis of Complexes ***3a,b***

A solution of the chloride anion-containing triphenylphosphine palladacycle adduct **2** (0.1 mmol) and NaI (0.2 mmol) in acetone (10 mL) was stirred at room temperature for 3h. The product was separated by passing through a silica gel column with dichloromethane as eluent. The first band was collected to afford the corresponding complex **3** after evaporation of the solvent.

[PdI{[(η^5^-C_5_H_5_)]Fe[(η^5^-C_5_H_3_)-N_2_C_4_H_2_-Cl]}PPh_3_] (**3a**): Yield 96%. Found (%): C, 48.7; H, 3.1; N, 3.8. Calc. (%) for C_32_H_25_ClFeIN_2_PPd: C, 48.5; H, 3.2; N, 3.5. IR (KBr, cm^−1^): 3043, 1582, 1533, 1470, 1433, 1392, 1374, 1268, 1158, 1091, 998, 838, 824, 749, 680. ^1^H-NMR (CDCl_3_): δ 7.85–7.81 (m, 6H, ArH), 7.44–7.38 (m, 11H, ArH), 4.57 (s, 1H, C_5_H_3_), 4.19 (s, 1H, C_5_H_3_), 3.70 (s, 5H, C_5_H_5_), 3.59 (s, 1H, C_5_H_3_). 

[PdI{[(η^5^-C_5_H_5_)]Fe[(η^5^-C_5_H_3_)-NC_5_H_3_-Br]}PPh_3_] (**3b**): Yield 97%. Found (%): C, 56.9; H, 3.9; N, 4.4. Calc. (%) for C_35_H_31_Cl_2_FeN_2_PPd: C, 56.5; H, 4.2; N, 4.7%. IR (KBr, cm^−1^): 3066, 2922, 1594, 1494, 1479, 1432, 1386, 1152, 1107, 1090, 1024, 998, 820, 746, 668. ^1^H-NMR (CDCl_3_): δ 9.87 (s, 1H, PyH), 7.83–7.78 (m, 6H, ArH), 7.75 (d, 1H, PyH), 7.45–7.39 (m, 9H, ArH), 7.17 (d, 1H, PyH), 4.57 (s, 1H, C_5_H_3_), 4.05 (s, 1H, C_5_H_3_), 3.65 (s, 5H, C_5_H_5_), 3.46 (s, 1H, C_5_H_3_). 

### 3.4. General Procedure for the Arylation Reactions

A prescribed amount of catalyst, aryl halide (0.5 mmol), phenyl boronic acid (0.75 mmol) or alkyne (0.6 mmol) and the selected base (1.5 mmol) in solvent (3 mL) were placed in a Schlenk tube under nitrogen. The reaction mixture was heated at 100 °C–120 °C for 12–24 h, then cooled and quenched with water. The reaction mixture was extracted three times with CH_2_Cl_2_, then the combined organic layers were washed with water, dried (MgSO_4_), and evaporated to dryness. The products were isolated by flash chromatography on silica gel using petroleum ether as eluent and analyzed by ^1^ H-NMR.

### 3.5. Crystal Structure Determination

Crystallographic data for complexes **2a** and **3a**,**b** were collected on a Bruker SMART APEX-II CCD diffractometer euuipped with a graphite monochromator at 296 K using Mo-Ka radiation (λ = 0.071073 Å). The data were corrected for Lorentz polarization factors as well as for absorption. Structures were solved by direct methods and refined by full-matrix least-squares methods on *F^2^* with the SHELX-97 program [31]. All non-hydrogen atoms were refined anisotropically, while hydrogen atoms were placed in geometrically calculated positions. Crystal data and structure refinements are summarized in Table 4. **CCDC** reference numbers 875474–875476 conta1n the crystallographyic data for **2a** and **3a**,**b**, respectively. These data can be obtained free of charge from The Cambridge Crystallographic Data Centre via www.ccdc.cam.ac.uk/ data_request/cif.

**Table 4:** Crystallographic data and structure refinement for **2a**–**3b**.

Compound	2a	3a	3b
Elemental formula	C_32_H_25_Cl_2_FeN_2_PPd	C_32_H_25_ClFeIN_2_PPd	C_33_H_26_BrFeINPPd
Formula mass	701.66	793.11	836.58
Crystal system	Monoclinic	Monoclinic	Triclinic
Space group	P2(1)/c	P2(1)/c	P-1
Crystal size/mm	0.38 × 0.27 × 0.16	0.35 × 0.27 × 0.18	0.34 × 0.26 × 0.17
a / Å	9.2765(7)	8.835(3)	10.410(3)
b / Å	15.8968(13)	16.756(6)	16.185(4)
c / Å	19.7419(16)	20.546(7)	17.981(4)
α / ^o^	90	90	85.597(3)
β / ^o^	90.8120(10)	90.789(4)	86.449(3)
γ / ^o^	90	90	81.452(3)
V / Å^3^	2911.0(4)	3041.4(18)	2983.0(12)
D_c_ / g cm^−3^	1.601	1.732	1.863
Z	4	4	4
Data/restraints/parameters	5419/0/352	5623/0/352	10997/0/703
R_1_, wR_2_ [I > 2σ(I)]^a^	0.0251, 0.0551	0.0595, 0.1755	0.0525, 0.1108
^a^ R_1_ = Σ||F_o_| − |F_c_|| / Σ|F_o_|, wR_2_ = [Σ (F_o_^2^ − F_c_^2^)^2^/ Σw(F_o_^2^)^2^]^ 1/2^			

## 4. Conclusions

In summary, we have prepared and characterized three new triphenylphosphine adducts of palladacycles containing chloride/iodide anions. Single-crystal X-ray analysis confirms that there are intermolecular C–H···X(Cl, Br, I) hydrogen bonds in the crystals of **2a**–**3b**. Their performance and the effect of halogen anions were evaluated in arylation reactions. Currently, further investigation into the mechanism of these reactions, and other applications involving this class of complexes in other coupling reactions are in progress. 
